# Left colon as a novel high-risk factor for postoperative recurrence of stage II colon cancer

**DOI:** 10.1186/s12957-020-01818-7

**Published:** 2020-03-11

**Authors:** Liming Wang, Yasumitsu Hirano, Toshimasa Ishii, Hiroka Kondo, Kiyoka Hara, Nao Obara, Shigeki Yamaguchi

**Affiliations:** Division of Gastroenterological Surgery, Saitama Medical University International Medical Center, Yamane, Hidaka-shi, Saitama, 350-1298 Japan

**Keywords:** Colorectal cancer, Carcinoembryonic antigen, Stage II, Left colon cancer

## Abstract

**Background:**

It is not clear whether stage II colon and rectal cancer have the same risk factors for recurrence. Thus, the purpose of this study was to identify the risk factors for postoperative recurrence in stage II colorectal cancer.

**Patients and methods:**

We retrospectively analyzed the data of 990 patients who had undergone radical surgery for stage II colorectal cancer. Patients’ pathological features and characteristics including age, sex, family history, body mass index, tumor diameter, gross type of tumor, infiltration degree (T3/T4), tumor grade, perineural invasion, vascular invasion, lymphatic invasion, pathologic examination of lymph node number, and preoperative carcinoembryonic assay (CEA) level was compared between patients with and without recurrence. Finally, the prediction of the left and right colons was analyzed.

**Results:**

The mean ages of the colon cancer and rectal cancer patients were 69.5 years and 66.4 years, respectively. In total, 508 (82.1%) and 285 (76.8%) patients were treated laparoscopically for colon cancer and rectal cancer, respectively, with median follow-up periods of 42.2 months and 41.8 months, respectively. Forty-four recurrences occurred in both the colon cancer (7.1%) and rectal cancer (11.9%) groups. The preoperative serum CEA level and T4 infiltration were significantly higher in recurrent colorectal cancer patients. The postoperative recurrence rate of left colon cancer (descending colon, sigmoid colon) was higher than that of right colon cancer (cecum, ascending colon, transverse colon) (OR 2.191, 95% CI 1.091–4.400, *P* = 0.027). In COX survival factor analysis of colon cancer, the left colon is one of the independent risk factors (risk ratio 5.377, 95% CI 0.216–0.88, *P* = 0.02). In disease-free survival (DFS), the left colon has a relatively poor prognosis (*P* = 0.05). However, in the COX analysis and prognosis analysis of OS, no difference was found between the left colon and the right colon.

**Conclusion:**

Preoperative CEA and depth of infiltration (T4) are high-risk factors associated with recurrence and are prognostic factors in stage II colorectal cancer. Left colon is also a risk factor for postoperative recurrence of stage II colon cancer.

Colorectal cancer (CRC) is one of the most common cancers and a leading cause of cancer-related death in men and women [1]. CRC is also a major cause of death in Japan, being the leading cause in women and the third most common cause in men [2]. The efficacy of adjuvant chemotherapy for stage II CRC remains controversial, although the benefit of adjuvant chemotherapy for stage III CRC has been established [3–6]. Many studies have reported that rectal cancer differs from colon cancer in etiology, genetics, clinical manifestation, anatomy, and biological characteristics [7], but it is unclear whether stage II colon and rectal cancer have the same risk factors for recurrence. The purpose of this study was to identify the risk factors for postoperative recurrence in stage II colorectal cancer.

## Patients and methods

We retrospectively analyzed the clinical data of 990 patients with stage II CRC in the Division of Gastroenterological Surgery of Saitama Medical University from 2007 to 2016. The surgery was considered therapeutic when there was no macroscopic or microscopic residual cancer after surgery. There were 579 men and 411 women, comprising 619 patients with colon cancer and 371 patients with rectal cancer. Patients receiving preoperative treatment or presenting with intestinal obstruction or perforation were excluded from the analysis.

Peripheral blood samples were collected before surgery. The serum CEA level was determined by radioimmunoassay. The CEA level was considered high at ≥ 5 ng/ml. The resected specimens were pathologically classified according to the 7th edition of the Union for International Cancer Control TNM classification of malignant tumors.

All patients underwent follow up with regular physical and blood examinations, colonoscopy, and computed tomography. All statistical analyses were performed using the SPSS software package version 22.0 for Macintosh (IBM Japan, Tokyo, Japan). The significance of the correlations between the preoperative CEA level and the pathological features was analyzed using the chi-squared test for independence according to each parameter. In order to control for confounding factors, binary logistic regression was used. Wald test was used to evaluate the significance of the association. Survival curves were plotted with the Kaplan–Meier method and analyzed with a log-rank test. *P* < 0.05 was considered statistically significant.

## Results

### Clinical characteristics of CRCs

As shown in Table 1, a total of 990 CRC patients were included, comprising 371 with rectal cancer and 619 with colon cancer. The mean ages of colon cancer and rectal cancer patients were 69.5 years and 66.4 years, respectively. Of these, 508 (82.1%) of the colon cancer patients and 285 (76.8%) of the rectal cancer patients were treated laparoscopically. The median follow-up periods were 42.2 months for colon cancer and 41.8 months for rectal cancer. Forty-four recurrences occurred in both the colon cancer (7.1%) and rectal cancer (11.9%) groups. We observed significant differences between the colon and rectal cancer patients regarding sex, average age, postoperative recurrence rate, gross type, serum CEA levels, and vascular invasion (all *P* < 0.05). Other features were not significantly different, including open or laparoscopic methods, differentiation, invasion depth (T), perineural invasion, cancer diameter, and infiltration of lymphatic vessels.

**Table 1:** Clinicopathological parameters in stage II colon and rectal cancer

Clinicopathological Parameters	Rectal cancer	Colon cancer	*P* value
Gender (Total n=)	371 (100.00%)	619 (100.00%)	
Male	245 (66.04%)	334 (53.96%)	
Female	126 (33.96%)	285 (46.04%)	<0.01
Age (year)	66.4±0.55	69.5±0.42	<0.01
Post Operation Recurrence			
No	327 (88.14%)	574 (92.73%)	
Yes	44 (11.86%)	44 (7.11%)	0.011
Open or Laparoscopic			
Open	86 (23.18%)	113 (18.26%)	
Laparoscopic	285 (76.82%)	508 (82.07%)	0.059
Gross Type			
Protruding	18 (4.85%)	56 (9.05%)	
Ulcerative & Infiltratie	353 (95.15%)	563 (90.95%)	0.015
Diameter (cm)	5.44±0.11	5.24±0.10	0.105
Differentiation			
Well & Moderate	354 (95.42%)	581 (93.86%)	
Poor & Mucinous	17 (4.58%)	38 (6.14%)	0.37
T			
T3	329 (89.648%)	536 (87.39%)	
T4	39 (10.51%)	78 (12.60%)	0.324
CEA (ng/mL)			
<5	192 (51.75%)	400 (64.62%)	
≥5	179 (48.25%)	219 (35.38%)	<0.01
Number of dissected lymph nodes			
<12	59 (15.90%)	71 (11.47%)	
≥12	312 (84.10%)	548 (88.53%)	0.05
Perineural Invasion			
No	316 (85.18%)	523 (84.49%)	
Yes	55 (14.82%)	96 (15.51%)	0.77
Vascular Invasion			
No	318 (85.71%)	263 (42.49%)	
Yes	53 (14.29%)	356 (57.51%)	<0.01
Infiltration lymphatic vessels			
No	312 (84.10%)	501 (80.94%)	
Yes	59 (15.90%)	118 (19.06%)	0.24

### Comparisons of clinicopathological parameters between CRC recurrence and non-recurrence

Rectal cancer recurrence was associated with a body mass index greater than 25 kg/m^2^ (*P* = 0.0001), a larger tumor size (6.00 ± 0.34; *P* < 0.001), advanced T stage (*P* < 0.005), higher serum CEA levels (*P* < 0.002), poor differentiation or mucinous histology (*P* < 0.007), perineural invasion (*P* < 0.001), vascular invasion (*P* = 0.028), and infiltration of lymphatic vessels (*P* < 0.001) (Table 2).

**Table 2:** Clinicopathological parameters for rectal cancer recurrence and non-recurrence

Clinicopathological Parameters	Rectal Non-Recurrence	Rectal Recurrence	*P* value
Gender (Total n=)	327 (100.00%)	44 (100.00%)	
Male	216 (66.06%)	29 (65.91%)	
Female	111 (33.94%)	15 (34.09%)	0.88
Age (year)	66.35±1.32	65.7±0.55	0.30
Cancer Familly History			
None	140 (42.81%)	22 (50.00%)	
Yes	187 (57.19%)	22 (50.00%)	0.45
BMI			
<25	309 (94.50%)	34 (77.27%)	
≥25	18 (5.50%)	10 (22.73%)	0.0001
Duplicate cancer			
None	298 (91.13%)	38 (86.36%)	
Yes	29 (8.87%)	6 (13.64%)	0.45
Multiple Cancer			
None	308 (94.19%)	38 (86.36%)	
Yes	19 (5.81%)	6 (13.64%)	0.104
Post Appendectomy			
None	266 (81.35%)	37 (84.09%)	
Yes	61 (18.65%)	7 (15.91%)	0.81
Gross Type			
Protruding	20 (6.12%)	2 (4.55%)	
Ulcerative & Infiltratie	307 (93.88%)	42 (95.45%)	0.94
Diameter (cm)	5.42±0.11	6.00±0.34	0.045
Differentiation			
Well & Moderate	316 (96.64%)	38 (86.36%)	
Poor & Mucinous	11 (3.36%)	6 (13.64%)	0.007
T			
T3	298 (91.15%)	30 (68.17%)	
T4	29 (8.85%)	14 (31.82%)	0.005
CEA (ng/mL)			
<5	187 (57.19%)	14 (31.82%)	
≥5	140 (42.81%)	30 (68.18%)	0.002
Number of dissected lymph nodes			
<12	54 (16.51%)	6 (13.64%)	
≥12	273 (83.49%)	38 (86.36%)	0.78
Perineural Invasion		(0.00%)	
No	49 (14.98%)	37 (84.09%)	
Yes	278 (85.02%)	7 (15.91%)	<0.001
Vascular Invasion			
No	118 (36.09%)	8 (18.18%)	
Yes	209 (63.91%)	36 (81.82%)	0.028
Infiltration lymphatic vessels			
No	291 (88.99%)	29 (65.91%)	
Yes	36 (11.01%)	15 (34.09%)	<0.001

Compared with rectal cancer, colon cancer recurrence was associated with just an advanced T stage (*P* < 0.0001) and higher serum CEA levels (*P* = 0.009) (Table 3).

**Table 3:** Clinicopathological parameters for colon cancer recurrence and non-recurrence

Clinicopathological Parameters	Colon Non-Recurrence	Colon Recurrence	*P* value
Gender (Total n=)	575 (100.00%)	44 (100.00%)	
Male	309 (53.74%)	24 (54.55%)	
Female	266 (46.26%)	20 (45.45%)	0.95
Age (year)	69.7±0.43	70.32±1.73	0.35
Cancer Familly History			
None	272 (47.30%)	19 (43.18%)	
Yes	303 (52.70%)	25 (56.82%)	0.71
BMI			
<25	448 (77.91%)	36 (81.82%)	
≥25	127 (22.09%)	8 (18.18%)	0.67
Duplicate cancer			
None	493 (85.74%)	40 (90.91%)	
Yes	82 (14.26%)	4 (9.09%)	0.46
Multiple Cancer			
None	543 (94.43%)	42 (95.45%)	
Yes	32 (5.57%)	2 (4.55%)	0.95
Tumor location			
Left colon	278 (48.34% )	27( 61.37%)	
Right colon	297 (51.65%)	17 (38.63% )	0.09
Gross Type			
Protruding	51 (8.87%)	1 (2.27%)	
Ulcerative & Infiltratie	524 (91.13%)	43 (97.73%)	0.21
Diameter (cm)	5.2±0.10	5.45±0.48	0.25
Differentiation			
Well & Moderate	527 (91.65%)	37 (84.09%)	
Poor & Mucinous	48 (8.35%)	7 (15.91%)	0.15
T			
T3	511 (88.8%)	30 (68.18%)	
T4	64 (11.2%)	14 (31.82%)	<0.0001
CEA (ng/mL)			
<5	380 (66.09%)	20 (45.45%)	
≥5	195 (33.91%)	24 (54.55%)	0.009
Number of dissected lymph nodes			
<12	68 (11.83%)	4 (9.09%)	
≥12	507 (88.17%)	40 (90.91%)	0.76
Perineural Invasion	(0.00%)		
No	494 (85.91%)	40 (90.91%)	
Yes	81 (14.09%)	4 (9.09%)	0.48
Vascular Invasion			
No	250 (43.48%)	13 (29.55%)	
Yes	325 (56.52%)	31 (70.45%)	0.1
Infiltration lymphatic vessels			
No	469 (81.57%)	35 (79.55%)	
Yes	106 (18.43%)	9 (20.45%)	0.89

### Correlations between the preoperative CEA levels and the site of recurrence

Local recurrences were significantly more common for rectal cancers with a higher CEA level than for those with a lower CEA level (*P* < 0.05). However, there was no significant difference in liver metastasis, lung metastasis, or peritoneal spread between the two groups (Table 4).

**Table 4:** Correlations between the preoperative CEA levels and the site of recurrence in stage II rectal cancer and colon cancer

	Rectal Cancer CEA ( ng/ml)			Colon Cancer CEA ( ng/ml)		
	CEA<5	CEA ≥5	*p*-Value	CEA <5	CEA ≥5	*p*-Value
	*n* = 191	*n* = 170		*n* = 389	*n* = 219	
Liver metastasis						
Negative	187 (97.91%)	161 (94.71%)		381 (97.94%)	209 (95.43%)	
Positive	4 (2.09%)	9 (5.29%)	0.10	8 (2.06%)	10 (4.57%)	0.07
Lung metastasis						
Negative	187 (97.91%)	163 (95.88%)		384 (98.71%)	214 (97.72%)	
Positive	4 (2.09%)	7 (4.12%)	0.26	5 (1.29%)	5 (2.28%)	0.35
Local recurrence						
Negative	185 (96.08%)	157 (92.35%)		384 (98.71%)	213 (97.26%)	
Positive	6 (3.92%)	13 (7.65%)	0.028	5 (1.29%)	6 (2.74%)	0.96
Peritoneal dissemination						
Negative	190 (99.48%)	169 (99.41%)		386 (99.23%)	213 (97.26%)	
Positive	1 (0.52%)	1 (0.59%)	0.93	3 (0.77%)	6 (2.74%)	0.053

Although the patients with higher CEA levels were more likely to develop colon cancer recurrence, there was no significant difference in the site of recurrence (Table 4).

### Correlations between the depth of infiltration and the site of recurrence

Local recurrences were significantly more common for colorectal cancers with T4 infiltration than for those with T3 infiltration (both *P* < 0.05) (Table 5). There was a higher rate of lung metastasis recurrence in patients with T4 rectal cancer compared with T3 (*P* = 0.004). In patients with T4 colon cancer, there was a higher rate of peritoneal metastasis (*P* = 0.014).

**Table 5:** Correlations between the depth of infiltration and the site of recurrence in stage II rectal cancer and colon cancer

	Rectal Cancer			Colon Cancer		
	T3	T4	*p*-Value	T3	T4	*p*-Value
	*n* = 332	*n* = 39		*n* = 542	*n* = 77	
Liver metastasis						
Negative	320 (96.39%)	38 (97.44%)		526 (97.05%)	74 (96.10%)	
Positive	12 (3.61%)	1 (2.56%)	0.735	16 (2.95%)	3 (3.90%)	0.651
Lung metastasis						
Negative	325 (97.89%)	35 (89.74%)		535 (98.71%)	74 (96.10%)	
Positive	7 (2.11%)	4 (10.26%)	0.004	7 (1.29%)	3 (3.90%)	0.089
Local recurrence						
Negative	318 (95.78%)	34 (87.18%)		534 (98.52%)	70 (90.91%)	
Positive	14 (4.22%)	5 (12.82%)	0.021	8 (1.48%)	7 (9.09%)	<0.001
Peritoneal dissemination						
Negative	331 (99.70%)	38 (97.44%)		538 (99.26%)	74 (96.10%)	
Positive	1 (0.30%)	1 (2.56%)	0.067	4 (0.74%)	3 (3.90%)	0.014

In multivariate analysis, a higher CEA level was associated with a higher chance of recurrence of both rectal cancer (odds ratio [OR] 1.011, 95% confidence interval [95% CI] 1.00–1.021, *P* = 0.048) and colon cancer (OR 1.010 95% CI 1.003–1.017, *P* = 0.004). In addition, T4 cancer had a higher chance of recurrence in both rectal cancer (OR 3.867, 95% CI 1.547–9.663, *P* = 0.004) and colon cancer (OR 3.222, 95% CI 1.238–8.390, *P* = 0.017) (Table 6). In colon cancer, the postoperative recurrence rate of left colon cancer (descending colon, sigmoid colon) was higher than that of right colon cancer (cecum, ascending colon, transverse colon) (OR 2.191, 95% CI 1.091–4.400, *P* = 0.027). However, there was no significant difference between low rectal cancer and upper rectal cancer.

**Table 6:** Multivariate logistic regression analysis evaluating possible risk factors associated with recurrence

	Rectal Cancer			Colon Cancer		
	Odds ratio	95% CI	*p*	Odds ratio	95% CI	*p*
Gender	1.259	0.587-2.70	0.554	0.879	0.453- 1.704	0.702
Age, year	0.983	0.953 -1.014	0.270	1.017	0.985-1.050	0.300
T4 vs T3	3.867	1.547-9.663	0.004	3.222	1.238-8.390	0.017
CEA (ng/mL)	1.011	1.000-1.021	0.048	1.010	1.003 -1.017	0.004
Tumor location	Rb vs Ra, RS			Left colon vs Right colon		
	0.825	0.411- 1.651	0.589	2.191	1. 091-4.400	0.027

Tumor location*: Rectal cancer (Rb & Ra, RS). *Ra* rectum above the peritoneal reflection, *Rb* rectum below the peritoneal reflection, *RS* rectosigmoid. Colon cancer: Left colon (Descending colon, sigmoid colon) & Right colon( Cecum, ascending colon, transverse colon)

### Correlations of the preoperative CEA levels and depth of infiltration with the survival rate

**Fig. 1:**
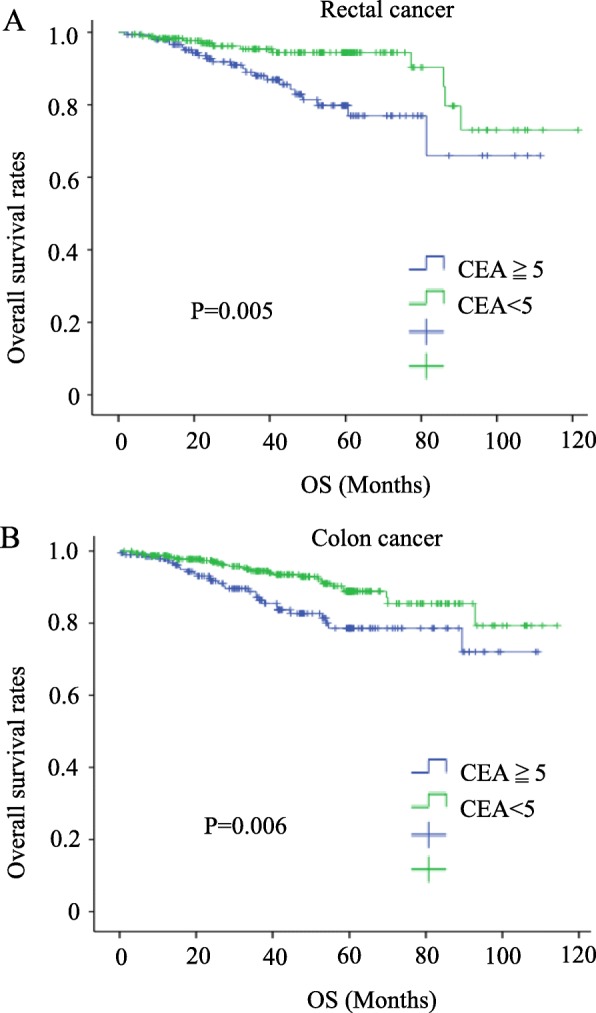
Survival outcomes for colorectal cancer patients with CEA ≧ 5 vs. CEA < 5

**Fig. 2:**
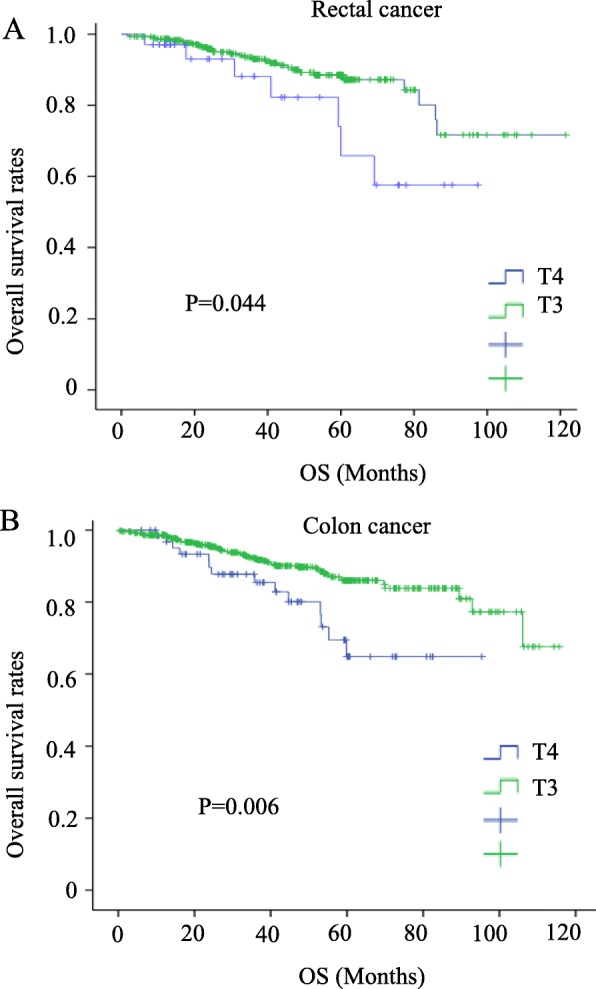
Survival outcomes for colorectal cancer patients with T3 vs. T4

**Fig. 3:**
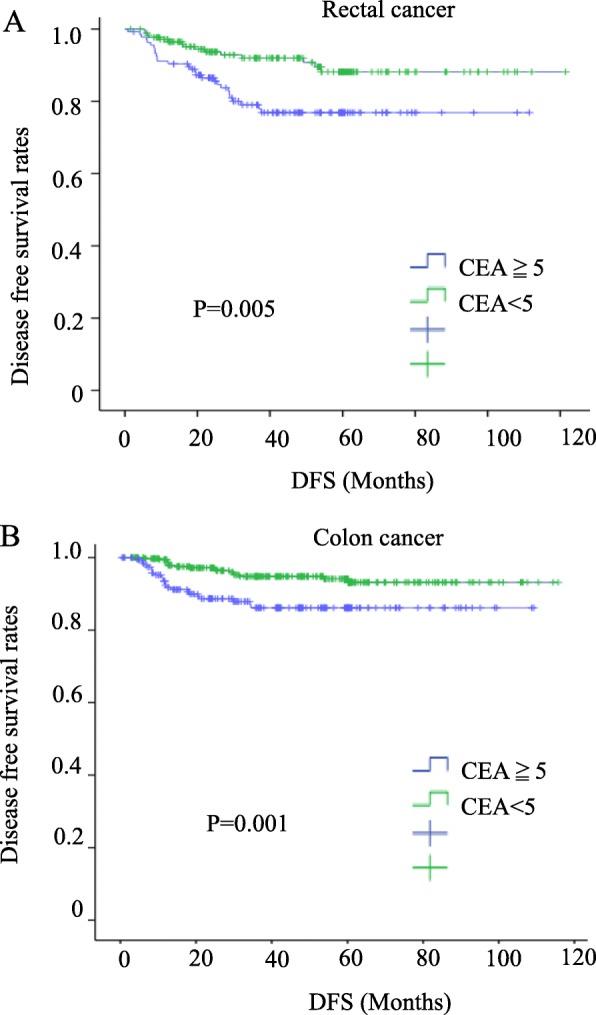
Disease-free survival for colorectal cancer patients with CEA ≧ 5 vs. CEA < 5

**Fig. 4:**
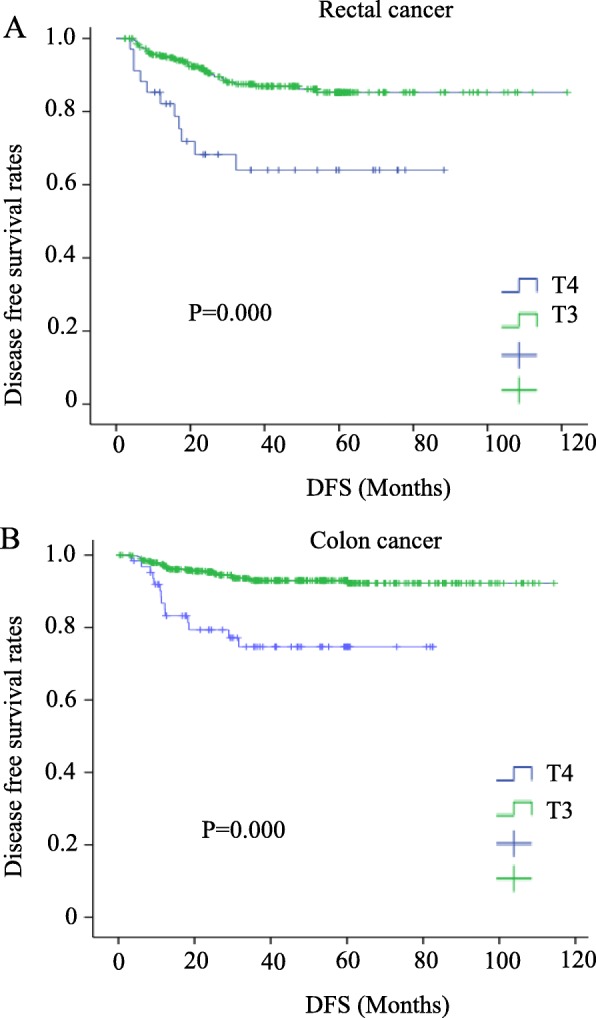
Disease-free survival for colorectal cancer patients with T3 vs. T4

The overall survival rate was significantly lower in both colon and rectal cancer patients with high levels of CEA and in T4 patients (*P* = 0.005, 0.006, 0.0044, and 0.006, respectively) (Figs. 1a, b and 2a, b). High levels of serum CEA and T4 reduced the disease-free survival (*P* = 0.005, 0.001, 0.000, and 0.000) (Figs. 3a, b and 4a, b).

### Prognostic differences between the left colon and the right colon

**Fig 5:**
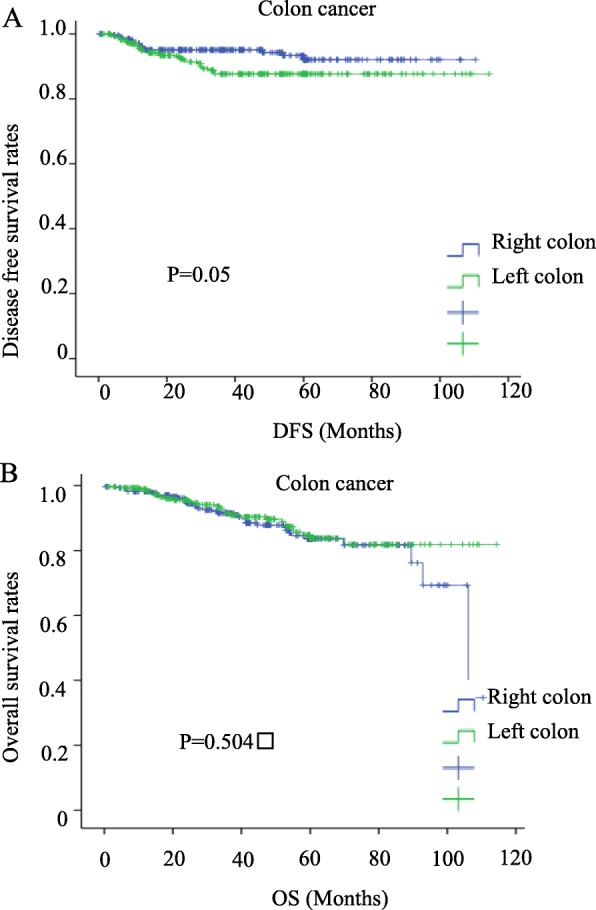
Prognosis of left colon cancer vs. right colon cancer

To compare the prognosis of histological parameters determined in univariate analysis, Cox’s proportional hazard regression model was applied. For DFS, left colon, vascular invasion, infiltrating pattern, and T4 were all shown to be independent risk factors. For OS, signet ring cell carcinoma, mucinous carcinoma, poorly differentiated carcinoma, vascular invasion, infiltrating pattern etc. were shown to be independent risk factors (Table 7). For the DFS Kaplan-Meier survival curve, the left colon also has a relatively poor prognosis. However, in the OS curve, there is no difference between the left colon and the right colon (Fig. 5a, b). There is no statistical difference in the ratio of T4:T3 (*P* = 0.337) and CEA ≥ 5 ng/ml (*P* = 0.32) in left colon cancer and right colon cancer (detailed data not shown).

**Table 7:**
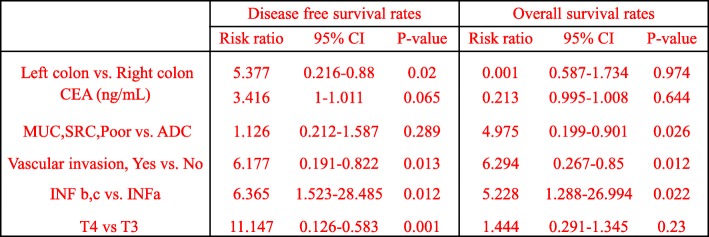
Cox proportional hazard regression model comparing the effects of different parameters on the prognosis of patients with stage II Colon cancer

*ADC* adenocarcinoma, *CEA* carcinoembryonic antigen, *CI* confidence interval, *SRC* signet ring cell carcinoma, *HR* hazard ratio, *Poor* poor poorly differentiated adenocarcinoma, *INF* Infiltrating pattern of invasion, *INFa* Swelling proliferation, *INF c* Invasive proliferation, *INF b* between a and c. *vs*. versus, Left colon(Descending colon, sigmoid colon); Right colon( Cecum, ascending colon, transverse colon)

## Discussion

The question of whether colon and rectal cancer should be treated as a single entity or as two separate entities remains controversial [8]. The treatment of stage II CRCs has also been extensively debated [7, 9]. Our findings indicate that these two groups show considerable differences in many clinic-pathological characteristics. Significant differences were seen between the two groups in sex, average age, postoperative recurrence, gross type, surgical procedure (open/laparoscopic), serum CEA level, and vascular invasion. Therefore, our results showed that, in stage II CRC, the characteristics of colon cancer and rectal cancer were different, which is why they should be considered two separate entities.

Out of 4,244 primary CRC patients, 990 cases (23.3%) of stage II CRC were found in our hospital in the past 10 years. In view of the different characteristics of rectal cancer and colon cancer, we independently analyzed their relapse characteristics. The depth of infiltration and level of the preoperative tumor marker CEA were directly related to recurrence in the colon cancer recurrence group compared with the non-recurrence group. This is consistent with the results of another study [10]. However, rectal cancer recurrence is much more complicated. It was not only related to the serum CEA level and depth of infiltration, but also obesity, tumor size, tumor type, and postoperative pathological lymphatic infiltration and perineural invasion. Therefore, CEA level and depth of infiltration are the common factors for the recurrence of the stage II CRC. These results are largely consistent with those of another study that also found that CEA and CA19-9 were factors associated with recurrence [11, 12].

We then looked at whether CEA was associated with the site of tumor recurrence. Although CEA is a complex glycoprotein that is the most commonly used tumor marker for CRC, it is highly nonspecific. In colon cancer, we did not find any relationship between CEA and the location of tumor recurrence but it was significantly associated with the local recurrence of rectal cancer. Local recurrence of rectal cancer involves lymph nodes near the sacrum, lymph nodes around the great arteries, and lateral lymph nodes. In this study, the preoperative CEA level and depth of infiltration (T4) were risk factors and prognostic factors for recurrence of stage II colon cancer. The different biological characteristics between rectal cancer and colon cancer lead to diverse recurrence factors and prognostic factors.

We found that the incidence of postoperative recurrence for left colon cancer was higher than that of right, and left colon is one of the independent risk factors of poorer DFS, which was consistent with some reports [13, 14]. The majority of the literature differs, reporting that there is no difference [15] or that the right colon has a relatively poor prognosis [16–19]. The inclusion of the rectum in the left colon is the biggest difference between these reports. This study also shows that many biological characteristics of the colon and rectum are different. The distinction between the descending colon and the sigmoid colon from the rectum may be more conducive to future treatment.

But more surprisingly, there was no difference in OS between the left colon and the right colon. This may be because the left colon, when compared with the right colon, even though RAS/BRAF are wild-type, has more advantages in the choice of chemotherapy drugs cetuximab or panitumumab [20]. It is important to recognize the limitations of this study. The chemotherapy effect of CRC is closely related to KRAS and BRAF gene mutations or microsatellite instability (MSI) [21]. In future analysis, research on genetic components needs to be strengthened. Second, this study is a single-center retrospective study with a relatively small sample size, and we look forward to future multi-center clinical studies.

## Conclusion

Our results indicate that the preoperative CEA level and depth of infiltration (T4) are high-risk factors associated with recurrence and are prognostic factors in stage II colorectal cancer. Left colon is also a risk factor for the postoperative recurrence of stage II colon cancer, and special attention should be paid during follow-up.

## Data Availability

Not applicable
